# Correlation of Hydronephrosis Index to Society of Fetal Urology Hydronephrosis Scale

**DOI:** 10.1155/2009/960490

**Published:** 2009-02-25

**Authors:** Krishnan Venkatesan, Joel Green, Steven R. Shapiro, George F. Steinhardt

**Affiliations:** ^1^Department of Urology, School of Medicine, Wayne State University, Detroit, MI 48201, USA; ^2^Department of Surgery, College of Human Medicine, Michigan State University, Grand Rapids, MI 49503, USA; ^3^Section of Urology, Sutter Memorial Hospital, Sacramento, CA, 95819, USA; ^4^Pediatric Urology Medical Group, Inc., Santa Monica, CA, 90403, USA; ^5^Department of Pediatric Urology, Helen DeVos Children's Hospital, Grand Rapids, MI 49503, USA

## Abstract

*Purpose*. We seek to correlate conventional hydronephrosis (HN) grade and hydronephrosis index (HI). *Methods*. We examined 1207 hydronephrotic kidneys by ultrasound. HN was classified by Society of Fetal Urology guidelines. HN was then gauged using HI, a reproducible, standardized, and dimensionless measurement of renal area. We then calculated average HI for each HN grade. *Results*. Comparing HI to standard SFU HN grade, average HI is 89.3 for grade I; average HI is 83.9 for grade II; average HI is 73.0 for grade III; average HI is 54.6 for SFU grade IV. *Conclusions*. HI correlates well with SFU HN grade. The HI serves as a quantitative measure of HN. HI can be used to track HN over time. Versus conventional grading, HI may be more sensitive in defining severe (grades III and IV) HN, and in indicating resolving, stable, or worsening HN, thus providing more information for clinical decision-making and HN management.

## 1. Introduction

Ultrasound (US) has gained widespread acceptance and use in fetal and pediatric urology. Hydronephrosis (HN) has become the most common abnormality detected by prenatal US [1]. HN had previously been characterized in a fairly subjective manner as mild, moderate, and severe. In 1993, the Society for Fetal Urology (SFU) established a grading system based on renal sinus splitting patterns and dilation of the renal pelvis and calyces [2]. Though the SFU grading system has been widely accepted, it does have certain deficiencies—especially in differentiation of severe (grades III and IV) HN [3]. Serial assessment of HN by US, often used in clinical decision making, relies on this grading system to suggest improving, stable, or worsening HN. Others have suggested improvements or complementary approaches to the SFU grading system [3–5]; none has gained widespread popularity of use.

Recently, a novel method to measure HN was suggested. The Hydronephrosis index (HI) has been shown to be a quantitative, reproducible, standardized measure of HN [6]. HI is calculated as a dimensionless number that represents renal area and can be used for serial examination of kidneys with HN. The present work serves to correlate the SFU grading system with the HI system, to advance familiarity with this new method of HN description.

## 2. Materials and Methods

An IRB approved prospective, computerized database accrued by the authors (George F. Steinhardt, Steven R. Shapiro) at their previous institution, was queried for all kidneys with the diagnosis of ureteropelvic junction obstruction (excluding kidneys with concomitant vesicoureteral reflux or sonographic ureteral dilation). All of these 1207 kidneys were then assessed using both the SFU grading system and the HI technique. The managing pediatric urologist (George F. Steinhardt) determined the SFU grade (I–IV) of HN; the pediatric radiologists, supervising the sonographic technician, determined HI. Studies were performed using an Acuson Sequoia 512 system (Siemens Medical Solutions, Malvern, Pa, USA).

**Figure 1:**
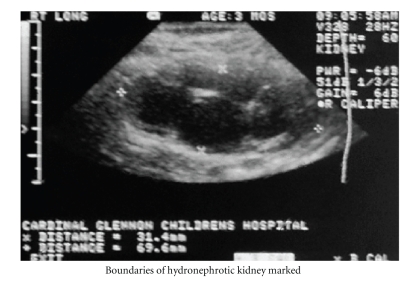
Boundaries of hydronephrotic kidney marked.

Specifically, a single sagittal US image was selected, where the kidney achieved its maximal longitudinal dimensions. The operator then marked the renal boundaries, traced an outline of the entire renal perimeter and then of the dilated renal pelvis (Figure 1). The portion of the renal pelvis extending beyond the kidney was not included in the HI [6]. The respective areas were then computed with integrated software which is standard on most modern ultrasound machines.

Data was entered into the database as patients were managed in the office.

HI is calculated as follows: HI (percentage) = 100 × (total area of the kidney minus area of dilated pelvis)/(total area). The result is a quantitative, dimensionless measurement of renal mass. In essence, HI represents the percentage of total kidney that is renal parenchyma.

For each grade of hydronephrosis, the average HI was calculated.

## 3. Results

The average HI was calculated for each group of kidneys, in order to establish a correlation of HI to HN grade (Table 1). The number of kidneys in each group is also listed in Table 1.

A normal nonhydronephrotic kidney would have an HI of 100. More hydronephrosis, or renal pelvis dilation, translates to a larger renal pelvic area and thus, a lower HI. For the group comprised of kidneys with grade I hydronephrosis, the average HI was 89.3. The group of grade II hydronephrotic kidneys had an average HI of 83.9. Kidneys with grade III hydronephrosis averaged 73.0 for HI. Average HI for grade IV hydronephrotic kidneys was 54.6.

The objectivity and reproducibility of HI measurements have been shown previously [6].

## 4. Discussion

Management of prenatal and infant HN relies heavily upon serial examination by US [7]. Currently, the main paradigm of HN description is the SFU grading system. The HI has been shown as a viable, alternate method of longitudinal HN monitoring [6]. It is an objective, reproducible, and standardized calculation. Because HI is quantitative and dimensionless, it allows easy portability of US interpretation across multiple locations and over time. The medical application of dimensionless numbers has been shown to be effective [8].

As one would expect, kidneys with more significant HN on average had a lower HI. While the difference in HI between lower grades of HN was minor, mean HI is markedly different at higher grades (III & IV) of HN. This end of the spectrum, differentiating between grades III and IV HN, is where the SFU grading system lacks clarity and depends heavily on individual interpretation [3]. The value of HI over the SFU grading system is the greatest in this regard. The subjective factor of visual interpretation is removed for one observer over time, as are discrepancies between multiple observers. In its place, an objective, quantitative interpretation of HN is produced.

**Figure 2:**
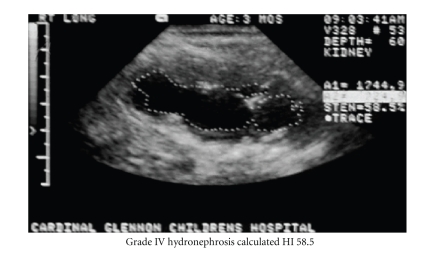
SFU grade IV hydronephrosis; Calculated HI 58.5. The dotted line outlines the renal pelvis [black] as used in calculating HI.

**Figure 3:**
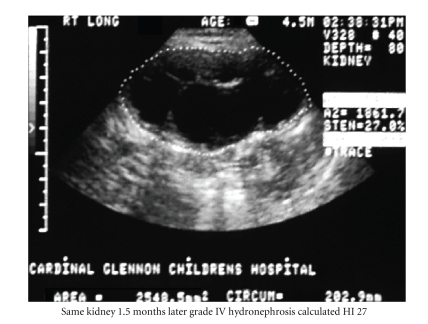
Same kidney 1.5 months later. SFU grade IV hydronephrosis; Calculated HI 27. The dotted line outlines the entire renal area, including renal pelvis [black] and parenchyma (dark gray). While the SFU grade remained the same, the HI has deteriorated significantly, indicating an actual worsening HN.

Serial measurement of HI may be more sensitive to subtle changes in hydronephrosis, not discriminated or perceived by the SFU system. Figures 2 and 3 demonstrate obvious changes in HN degree in a kidney with static SFU grade. This sensitivity also holds true for postoperative monitoring, where the SFU system may not detect results appropriately [5].

The quantified HI measurement serves as a “continuous” variable, in contrast to a discrete grade encompassing a wide range of pathology. This can affect better informed clinical decision making for a wide variety of hydronephrotic kidneys with diverse clinical settings. Accordingly, results of hydronephrosis management may improve as well. For example, earlier correction of UPJ obstruction has been shown to give better drainage [9]. Also, it is clear that glomeruli are irreparably damaged prior to any evident loss of GFR [10], suggesting that waiting longer for loss of function to manifest before intervening would be less than ideal. Therefore, worsening hydronephrosis constitutes a relative indication for surgery, and our technique better discriminates subtle changes in HN.

This work reviews the HI of 1207 renal units with HN to facilitate establishment of context for HI. Clinicians can now calculate and use HI with the confidence that it provides an objective, quantifiable interpretation for the management of HN. It correlates well with, and improves upon, the SFU grading system for HN and is especially valuable in management of high-grade HN.

The concept of quantification of HN has been addressed previously. Rodríguez et al. [4] described a series of 81 patients, in which they calculated renal parenchymal and pelvic areas, in a manner similar to our own. However, their technique differs in that they propose a computation to derive a threshold ratio which predicts the need for a surgery. This work, on the other hand, emphasizes HI as a more sensitive indicator of HN over time, not necessarily as a singular criterion for, or prognosticator of, surgery.

HI does have its limitations. The data (Table 1) shows that a wide HI range represents each SFU grade. For example, the lowest HI for grade I HN is 42, which is obviously quite low. One inherent problem in a dataset containing greater than 1200 renal units is the difficulty in accounting for data entry problems.

The standard deviation is calculated for each HI and bolsters the constructed framework. While there is minimal overlap of HI at lower grades of HN, on a larger spectrum, and the predicted trend is seen. While it may not be used reliably to compare different patients with HN, the calculated average HI does provide a reference point for better informed individual HN management.

Also, it is possible that some HI measurements were made with a full bladder. As this work is extracted from a database, original imaging was unavailable for review. However, this should have been acknowledged and accounted by the clinician at the time of HI review.

## 5. Conclusion

HI correlates predictably with the SFU grading system for HN. Because it provides a quantitative measurement, it can be used to predict the SFU HN grade. HI can also be employed for longitudinal monitoring of HN by US, including pre- and postoperative observation. Because it is a more sensitive indicator of renal parenchymal status, HI allows for better informed clinical decision making, identifying changes in HN not discerned by the current SFU system.

## Figures and Tables

**Table 1:** Correlation of HI to HN grade.

SFU grade	Mean HI	Range of HI	HI std dev	Number of kidneys
I	89.3	42–97	5.9	202
II	83.9	39–100	7.2	444
III	73.0	43–97	8.6	298
IV	54.6	20–94	12.5	263

## References

[1] Mandell J, Blyth BR, Peters CA, Retik AB, Estroff JA, Benacerraf BR (1991). Structural genitourinary defects detected in utero. *Radiology*.

[2] Fernbach SK, Maizels M, Conway JJ (1993). Ultrasound grading of hydronephrosis: introduction to the system used by the Society for Fetal Urology. *Pediatric Radiology*.

[3] Onen A (2007). An alternative grading system to refine the criteria for severity of hydronephrosis and optimal treatment guidelines in neonates with primary UPJ-type hydronephrosis. *Journal of Pediatric Urology*.

[4] Rodríguez LV, Lock J, Kennedy WA, Dairiki Shortliffe LM (2001). Evaluation of sonographic renal parenchymal area in the management of hydronephrosis. *The Journal of Urology*.

[5] Imaji R, Dewan PA (2002). Calyx to parenchyma ratio in pelvi-ureteric junction obstruction. *BJU International*.

[6] Shapiro SR, Wahl EF, Silberstein MJ, Steinhardt G (2008). Hydronephrosis index: a new method to track patients with hydronephrosis quantitatively. *Urology*.

[7] Belarmino JM, Kogan BA (2006). Management of neonatal hydronephrosis. *Early Human Development*.

[8] Wahl EF, Lerman SE, Lahdes-Vasama TT, Churchill BM (2004). Measurement of bladder compliance can be standardized by a dimensionless number: clinical perspective. *BJU International*.

[9] Chacko J, Boucat HAP, Dewan PA (1997). Improved drainage following early pyeloplasty for pelvi-ureteric junction obstruction. *Asian Journal of Surgery*.

[10] Chevalier RL, Gomez RA, Jones CE (1988). Developmental determinants of recovery after relief of partial ureteral obstruction. *Kidney International*.

